# State-dependent reactivity of anterior cingulate cortex neurochemistry and downstream autonomic arousal in intrusive thinking

**DOI:** 10.1007/s00702-025-02992-2

**Published:** 2025-09-04

**Authors:** Martino Schettino, Chiara Parrillo, Simone Gazzellini, Luca Cairone, Giulia Baldassari, Julian F. Thayer, Federico Giove, Antonio Napolitano, Cristina Ottaviani

**Affiliations:** 1 Department of Psychology, Sapienza University of Rome, Rome, Italy; 2 IRCCS Istituto Delle Scienze Neurologiche di Bologna, Bologna, Italy; 3 Bambino Gesù Children’s Hospital, Rome, Italy; 4 Department of Psychological Sciences, University of California, Irvine, CA USA; 5 Centro di Studi e Ricerche Enrico Fermi, Rome, Italy; 6 IRCCS Santa Lucia Foundation, Rome, Italy

**Keywords:** Perseverative cognition, Repetitive negative thinking, Generalized anxiety, Heart rate variability, Magnetic resonance spectroscopy, Resting state functional connectivity

## Abstract

Unwanted intrusive thoughts play a key role in the onset, maintenance, and relapse of stress-related psychopathological conditions and are usually accompanied by a physiological fight-or-flight response. However, the mechanisms underlying the persistence of this maladaptive process remain unclear. This study employed a multimodal approach, integrating proton magnetic resonance spectroscopy, resting-state functional connectivity, and heart rate monitoring, to investigate gamma-aminobutyric acid (GABA) and glutamate-mediated neurometabolism during intrusive thinking, alongside central and peripheral autonomic nervous system activity. In individuals meeting diagnostic criteria for Generalized Anxiety Disorder (*n* = 29), an experimental induction of intrusive thoughts was associated with an increase in anterior cingulate cortex (ACC) GABA+ (macromolecular-contaminated), which correlated with suppressed autonomic reactivity. This relationship was mediated by the dispositional tendency to engage in intrusive thinking and was accompanied by reduced resting-state activation of the central autonomic network. Conversely, age- and sex-matched healthy controls (*n* = 29) exhibited a reduction in ACC GABA + and increased autonomic reactivity during intrusive thinking. While limited by a cross-sectional design, these findings suggest that pathological intrusive thinking may be maintained through neurometabolic negative reinforcement mechanisms involving both the central and autonomic nervous systems. If corroborated, current results may inform the development of targeted treatments aimed at disrupting such reinforcement processes.

**Supplementary Information:**

The online version contains supplementary material available at 10.1007/s00702-025-02992-2.

## Introduction

Intrusive thinking, also known as perseverative cognition or repetitive negative thinking, is a debilitating transdiagnostic phenomenon characterized by intrusive, unwanted, repetitive, and uncontrollable thoughts (e.g., Shihata et al. 2022; Spinhoven et al. 2015a; Zagaria et al. 2023). Critically, it is a well-established predictor of the onset, maintenance, and relapse of stress-related psychopathological conditions (e.g.Nolen-Hoeksema 1991, 2000; Spinhoven et al. 2015b, 2018a; Taylor and Snyder 2021).

Substantial evidence indicates that intrusive thinking elicits a physiological stress response comparable in magnitude to that triggered by an actual stressor (Ottaviani et al. 2016 for a meta-analysis). During episodes of intrusive thoughts or images, the body responds as though it were confronting the perceived threats encoded in these mental representations (*Perseverative Cognition Hypothesis*; Brosschot et al. 2006). This chronic activation of the physiological *fight-or-flight* response, driven by the repetitive nature of intrusive thinking, contributes to allostatic load, rendering individuals psychologically and somatically vulnerable (Larsen et al. 2009). For instance, individuals with higher levels of intrusive thinking, regardless of diagnostic classification, face an elevated risk of developing cardiovascular disorders (Holman et al. 2008; Kubzansky et al. 1997; Seldenrijk et al. 2015; Tully et al. 2013).

Despite its clinical importance, current treatment strategies for intrusive thinking remain suboptimal (e.g., Bell et al. 2023; Spinhoven et al. 2018b). This limitation likely stems from an incomplete understanding of the neural mechanisms underlying its persistence. While progress has been made in identifying brain activation patterns and functional connectivity correlates (Demnitz-King et al. 2021; Makovac et al. 2020), the neurochemical basis of intrusive thinking remains poorly characterized.

A defining feature of intrusive thinking is the inability to suppress unwanted thoughts or images, highlighting the potential role of GABA, the brain’s primary inhibitory neutrotransmitter of the central nervous system. Evidence from preclinical studies (Brambilla et al. 2003) and postmortem analyses (e.g., Bielau et al. 2007; Rajkowska 2014) suggests altered GABA neurotransmission in stress-related disorders. Proton magnetic resonance spectroscopy (^1^H-MRS) offers a non-invasive reliable method for quantifying in vivo levels of GABA and glutamate proxies in individuals with psychopathological conditions, such as GABA + and Glx (glutamate + glutamine, Duda et al. 2021; Port 2020; Schür et al. 2016 for a meta-analysis). MRS studies have revealed significant changes in cortical GABA + in stress-related disorders such as anxiety, depression, and post-traumatic stress disorder (e.g., Long et al. 2013; Rosso et al. 2022; Xie et al. 2023). Furthermore, therapeutic agents targeting GABAergic (such as SAGE-217; e.g., Gunduz-Bruce et al. 2019) and glutamatergic neurotransmission (such as ketamine and D-cycloserine; e.g., Grunebaum et al. 2018) show promise for these conditions (reviewed in Boucherie et al. 2023).

However, existing studies largely focus on trait differences in baseline neurometabolite levels between individuals with psychopathology and healthy controls, with limited attention to state-dependent changes in GABA + levels following stress induction. Preclinical research demonstrates stress-induced alterations in cortical GABA-A receptor expression and GABAergic interneuron activity (Matsumoto et al. 2007; Moghaddam 2002; Shepard et al. 2018; Treccani et al. 2014). MRS studies in non-human primates further reveal long-lasting reductions in the GABA/Creatine ratio in the ACC following early life stressors (Mathew et al. 2003).

Few MRS studies have explored the effects of psychological stress on state-dependent GABA and glutamate proxies’ levels. For example, Hasler and colleagues reported an 18% decrease in medial prefrontal cortex (mPFC) GABA + levels during a threat-of-shock condition (Hasler et al. 2010), while Houtepen and colleagues observed no significant changes following the Trier Social Stress Test (Houtepen et al. 2017). Cooper and colleagues found that reduced mPFC Glx responses were negatively correlated with subjective stress levels during the Maastricht acute stress task, but this effect was absent in individuals with major depressive disorder (Cooper et al. 2021). These inconsistencies may arise from variability in perceived stress induced by standardized laboratory stressors. In our view, intrusive thinking about personally relevant stressors could address this limitation (Ottaviani 2018).

To date, only one study has examined the effects of intrusive thinking on GABA metabolites, reporting a positive association between resting hippocampal GABA levels, inhibitory control, and fronto-hippocampal coupling during unwanted thought suppression in healthy individuals (Schmitz et al. 2017). While hippocampal neural activity may be relevant to understand the mechanisms underlying thought suppression, a recent meta-analysis pointed to the anterior cingulate cortex (ACC) as a key prefrontal region to distinguish between pathological and non-pathological forms of intrusive thinking (Makovac et al. 2020). The ACC has also been consistently implicated in affective disorders such as depression, anxiety, and stress related conditions (Drevets et al. 2008), as well as in the experience of intrusive thoughts more specifically (e.g., Demnitz-King et al. 2021). Critically, the ACC forms part of the central autonomic network (CAN), a neural circuit responsible for regulating autonomic and cardiovascular response to internal or external stimuli (Benarroch 1993). In fact, functional neuroimaging studies have shown that ACC activity is associated with individual differences in vagally mediated heart rate variability (HRV), a proxy of parasympathetic nervous system function (e.g., Critchley et al. 2003; Tomasi et al. 2024). Notably, reduced HRV is a well-established physiological correlate of intrusive thinking, observed both as a trait and during experimentally induced intrusive thought states (Ottaviani et al. 2016 for a meta-analysis). Crucially, preclinical evidence further supports a mechanistic role for prefrontal neurotransmission in autonomic regulation: enhancing GABAergic activity in the ACC through administration of GABA receptor agonists reduces HR and increases HRV (Wallis et al. 2017), whereas glutamatergic hyperactivation leads to HRV reduction and induces anxiety-like responses (heightened intolerance to uncertain threat) in marmosets (Alexander et al. 2020). Lastly, the CAN has extensive inhibitory connections with the amygdala, insula and brainstem autonomic nuclei via intercalated GABAergic neurons (Thayer and Lane 2009).

In light of the reviewed rationale linking neurochemical changes in the ACC to autonomic regulation and the subjective experience of intrusive thoughts, the present study integrates ¹H-MRS, HRV analysis, and resting-state functional connectivity (RSFC) of CAN nodes to investigate their underlying neurochemical correlates.

We hypothesized that state-dependent changes in ACC GABA and glutamate following the experimental induction of intrusive thoughts would be associated with reduced autonomic regulation, as indicated by decreases in HRV and CAN RSFC from pre- to post-induction. Using a dimensional framework and a symptom-specific assessment, we recruited individuals with both pathological and non-pathological levels of intrusive thinking to examine potential group differences in ACC GABA and Glx neurometabolism at rest and during the induction.

## Methods

### Participants

Medication-free individuals were recruited from university psychological services and classes through flyers, internet postings, and word of mouth. We grouped individuals based on their scores on the Penn State Worry Questionnaire (PSWQ; Meyer et al. 1990), as it has a validated clinical cut-off. Participants with scores above 50 were assigned to the pathological intrusive thinking group (*n* = 29; 21.62 ± 3.19 years), while those with scores of 50 or below were assigned to the non-pathological control group (*n* = 29; 21.41 ± 2.16 years). Sample size was informed by the simulation study conducted by Desmond and Glover (2002), which estimated that approximately 24 participants per group are needed to achieve 80% power at the single-voxel level in fMRI studies, assuming a corrected *α* = 0.05.

Exclusion criteria included contraindication for an MRI exam, age below 18 years, severe somatic diseases, history of substance or alcohol abuse or dependence, obesity (body mass index > 30 kg/m^2^), medication intake, and pregnancy. All participants were native Italian speakers. Five participants (3 pathological, 2 non-pathological) were excluded due to psychopharmacological treatment at the time of the study. Participants received financial compensation (30 Euros) upon study completion. The study adhered to the declaration of Helsinki and approved by the Ethical Committee of IRCCS Santa Lucia Foundation (CE/PROG.896), with all participants providing written informed consent.

### Questionnaires

Participants completed questionnaires assessing socio-demographic and lifestyle information (nicotine and caffeine consumption, alcohol use and physical activity). They also completed measures of post-traumatic stress symptoms (Impact of Events Scale-Revised (IES-R; Sundin and Horowitz 2002), depression (Center for Epidemiologic Studies Depression Scale; CES-D; Radloff 1977), anxiety (State-Trait Anxiety Inventory (STAI-Y; Spielberger et al. 1970); 7-item Generalized Anxiety Disorder Scale (GAD-7; Spitzer et al. 2006) and Generalized Anxiety Disorder Questionnaire-IV (GAD-Q-IV, Newman et al. 2002)), and the tendency to engage in various patterns of intrusive thoughts (Rumination Response Scale (RRS (Treynor et al. 2003); Obsessive-Compulsive Inventory Revised (OCI-R; Foa et al. 2002)).

### Experimental design

**Fig. 1:**
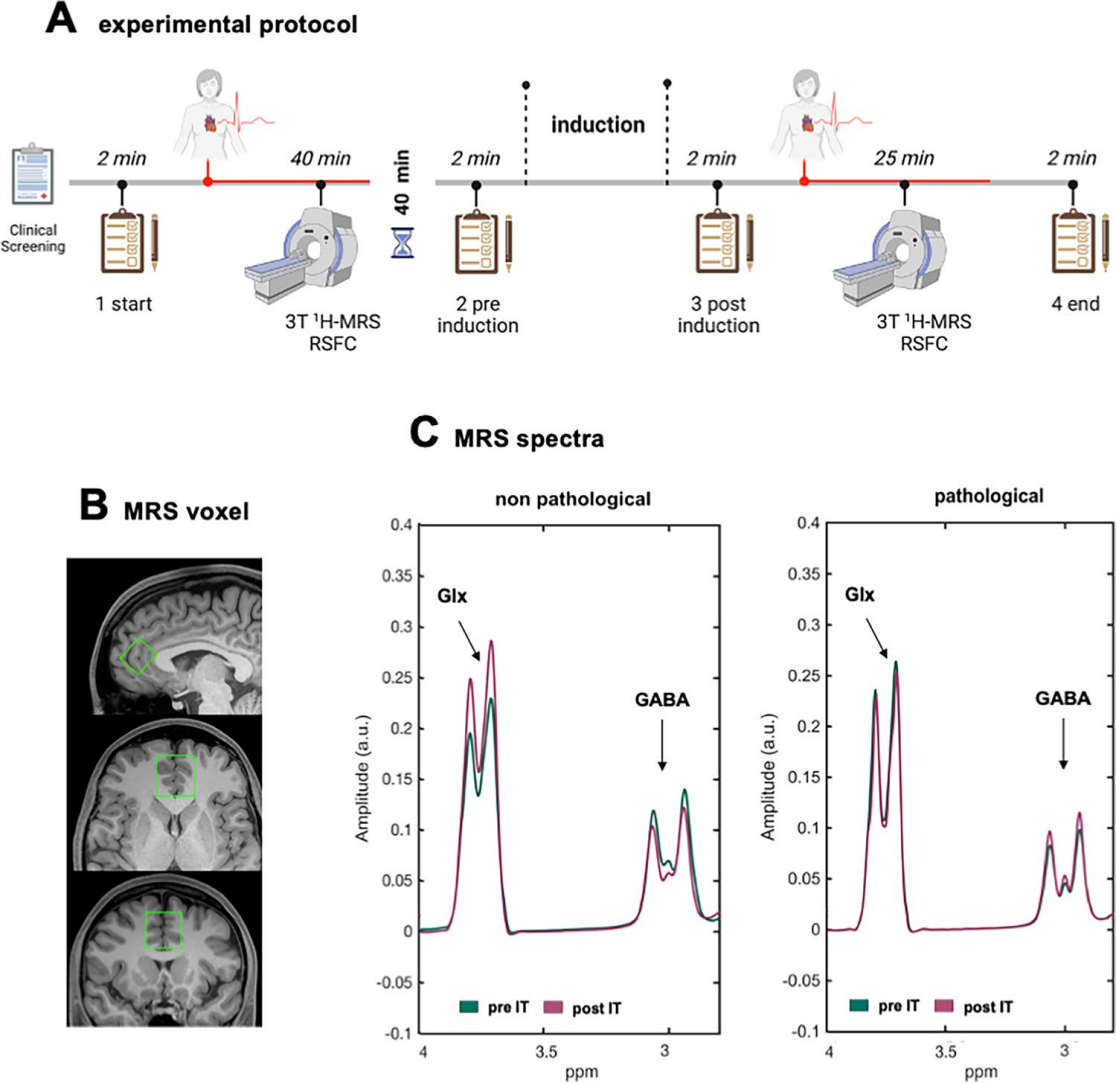
Layout of the experimental protocol (A). The clipboards represent visual analog scales (VASs) administered at four time points: before (baseline) and after the first scanning session (pre-induction of intrusive thinking), and before and after the second scanning session (post-induction). Magnetic Resonance Spectroscopy (MRS) voxel of interest (green) in the bilateral anterior cingulate cortex (ACC) is shown in the sagittal, axial, and coronal planes (B). Example of single-subject MRS spectra for one pathological and one non-pathological individual, separately shown for pre- and post-induction conditions in the ACC (MEGA-PRESS, 32×2 transients per condition); scale in parts per million (p.p.m.) (C). Resonances attributed to GABA and Glx are labeled.Note. 3T ¹H-MRS = 3 Tesla proton magnetic resonance spectroscopy; RSFC = resting-state functional connectivity; Pre IT = before the induction of intrusive thinking; Post IT = after the induction of intrusive thinking; Pathological = subsample characterized by a dispositional pathological tendency to engage in intrusive thinking; Non-pathological = subsample characterized by a low dispositional tendency to engage in intrusive thinking

Participants were asked to abstain from nicotine, caffeinated beverages, alcohol, heavy meals, and strenuous exercise for at least 48 hours prior to the experimental session. In the MRI scanner, participants rested with their eyes open and remained awake. Target metabolite concentration, whole brain functional connectivity, and autonomic measures (HR, HRV) were acquired at rest, both before and after the induction of intrusive thinking (Fig. 1A). Pre- and post-induction sessions were separated by a 40-min break to avoid fatigue and sleepiness. Each scanning block (pre, post) included a high-resolution structural scan, MRS, and RSFC acquisition (Fig. 1A). The intrusive thinking induction occurred immediately after the break in a dedicated room adjacent to the MRI scanner.

### Intrusive thinking induction and assessment

Using a well-replicated procedure effective in eliciting intrusive thinking in both clinical and non-clinical samples (reviewed in Ottaviani 2018), participants were instructed as follows: *“Next*,* I would ask you to recall an episode from the past or an anticipated future event that often intrudes in your mind. Please*,* take as much time as needed to recall it in detail*,* including its possible causes*,* consequences*,* and your feelings about it. Press the button whenever you are ready. Now*,* I ask you to tell me about the episode for 2 minutes.”*

To assess the state level of intrusive thinking, participants rated four 100-point Visual Analogue Scales (VASs): “Right now, how much are: 1) you distracted by your thoughts (i.e., past memories, future worries, personal problems)? (*absent-mindedness*); 2) these thoughts going through your mind again and again? (*repetitiveness*); 3) these thoughts coming to your mind without you wanting them to? (*intrusiveness*); 4) you stuck on these issues and unable to move on? (*stuckness*);” These VASs were administered at four time points: before (baseline) and after the first scanning session (pre-induction), and before (post-induction) and after the second scanning session (end of protocol).

### Physiological data acquisition and pre-processing

Inter-beat intervals were assessed using the scanner pulse oximeter to derive HR and HRV. Cardiac traces were sampled at 400 Hz and analyzed using Kubios HRV software (Tarvainen et al. 2014), which detected outliers and artifacts (using the automatic correction option) and performed HRV analyses. HRV was assessed by computing the root mean square of successive differences (RMSSD), reflecting vagal regulation of HR (Task Force 1996).

### MR images acquisition

MR images were collected using a 3 T scanner (Siemens MAGNETOM Prisma, Syngo version VE11E), equipped with a 32-channel head coil and high-power shim amplifiers. Transmit was via a 2-channel body coil. A T_1_-weighted structural scan was acquired during the pre-induction session using the MPRAGE (Magnetization-Prepared RApid Gradient-Echo) sequence (TE/TR/TI 2.8/2500/1170 ms, GRAPPA 2, flip angle 8°, isotropic voxel 0.8^3^ mm^3^). A T_2_-weighted structural scan was acquired during the post-induction session using SPACE (Sampling Perfection with Application optimized Contrast using different flip angle Evolution) (TE/TR 564/3200 ms, GRAPPA 2, variable flip angle, isotropic voxel 0.8^3^ mm^3^). To check for potential gross motion during the second session, a highly accelerated T_1−_weighted volumetric scan was also acquired after spectroscopy using CAIPIRINHA (Controlled Aliasing in Parallel Imaging Results in Higher Acceleration) (TE/TR 14/580 ms, acceleration 2 × 2, variable flip angle, isotropic voxel 1.0^3^ mm^3^). MPRAGE and SPACE scans included prospective motion correction and selective reacquisition of motion-corrupted data (Tisdall et al. 2012).

Functional scans were acquired using a T_2_* -weighted gradient-echo EPI sequence, with a multiband factor of 8, and an isotropic voxel size of 2.2^3^ mm^3^ (64 slices; field of view: 208 × 208 mm^2^). TE/TR was 32/800 ms, with a Flip Angle of 52° and no in-plane acceleration (Moeller et al. 2010; Xu et al. 2013). For each session, two runs were acquired with opposite phase encoding in the anterior-posterior direction, each consisting of 450 measurements. Single band reference images were obtained during the pre-scan. Two Spin echo scans without multiband and the same geometrical and sampling properties were acquired with opposite phase encoding direction for field mapping (TE/TR: 80/7660 ms; Flip angle: 90°).

### ^1^H-MRS data acquisition and analysis

GABA-edited single voxel localized spectroscopy on the ACC was performed with a highly optimized MEGA-PRESS (MEshcher-GArwood Point RESolved Spectroscopy) sequence (Marjańska et al. 2013; Mescher et al. 1996, 1998). The spectroscopic voxel (20 × 30 × 25 mm^3^, RLxAPxFH) was prescribed on the volumetric scan acquired during the session (either MPRAGE or SPACE) and adjusted for each participant to keep the anterior voxel face parallel to the skull (Fig. 1B), using standard anatomical guidance and minimaxing cerebrospinal fluid inclusion. A screenshot of the voxel placement of the pre-induction session was used to prescribe the voxel in the post-induction session.

MRS data processing followed a custom pipeline (detailed in S1). Preprocessing was performed in Gannet 3.0 toolkit (Edden et al. 2014), an open-source software coded in MATLAB (The MathWorks, Inc., Natick, MA, USA). Quantitation was performed using LCModel (Provencher 1993) with a simulated basis set.

Quality of the MRS data was quantitatively assessed using GABA + and Glx fit error, signal-to-noise ratio and frequency drift (reflected by the standard deviation of the frequency offset). LCmodel quantifications with Cramér-Rao lower bounds above 30% were discarded.

GannetCoRegister and GannetSegment modules, incorporating routines from SPM12 (Ashburner and Friston 2005), were used to determine tissue fractions (grey matter (GM), white matter (WM) and cerebrospinal fluid (CSF) within each voxel and time point. Raw concentrations were corrected for GM/WM metabolite distribution ratios (Harris et al. 2015), setting a correction factor of 0.5. The ratio between GABA + and Glx was computed as an indicator of the balance between inhibitory and excitatory (“I/E” balance) neurometabolic processes in the brain as reported in previous MRS studies (Rideaux et al. 2022). All metabolite values are reported in institutional units (i.u.) (Fig. S1 for the plotted spectra metabolites).

### Resting-state functional MRI data preprocessing and analyses

Functional data preprocessing was performed using the minimal preprocessing pipelines for the Human Connectome Project (HCP) (Glasser et al. 2013) as implemented in the open-source Quantitative Neuroimaging Environment & Toolbox (QuNex) software suite (Ji et al. 2023) (detailed in S2).

RSFC analyses focused on the CAN network, identified using the Hammersmith atlas (Hammers et al. 2003). Seven nodes were selected: dorsal, perigenual, and subgenual ACC, central nucleus of the amygdala, nucleus of solitary tract, medulla, and anterior insula. Pearson’s correlations between pairs of nodes time series were calculated as the standard measure of RSFC; correlation values were z-score normalized using Fisher transformation and thresholded at 0.2.

### Statistical analysis

Data are expressed as means (± SD). Preliminary evaluations ensured no violation of normality, linearity, homogeneity of variances, or sphericity. To test for pre-existing group differences, *t* and *χ*^*2*^ tests were conducted on self-report, sociodemographic, physiological, and dispositional measures.

To assess the effects of the induction on self-report, autonomic and neurochemical variables, General Linear Models (GLM) were performed on VAS, HR, RMSSD, GABA+, Glx, and GABA+/Glx considering *Condition* (pre- vs. post-induction) and *Group* (pathological vs. non-pathological) as factors. Significant Condition x Group interactions were followed by simple main effects analysis comparing pre- vs. post-induction differences within the pathological and non-pathological groups. Reactivity scores ((post-pre)/pre) were computed for GABA+, Glx, and GABA+/Glx, with group differences tested using a series of independent *t-*tests.

To control for multiple comparisons within each family of analyses, a Bonferroni correction was applied. A result was considered statistically significant if its *p*-value was below the adjusted threshold (*α/K*), where *α* was set at 0.05 and *K* represented the number of comparisons within that family (e.g., *α/K* = 0.025 for two comparisons).

Lastly, a multivariate analysis of covariance (MANCOVA) evaluated differences in pre-to post-induction changes ((post-pre)/pre) in RSFC within nodes of the CAN between groups, controlling for biological sex. When the overall *F*-test was significant, post-hoc MANCOVA identified specific node contributions to the model.

## Results

### Baseline differences

No significant differences were found between the pathological and control groups in sex, age, education, body mass index, nicotine use, or alcohol use. However, the pathological group engaged in fewer hours of habitual physical exercise than controls (Table 1).

**Table 1:** Socio-Demographic, clinical and baseline characteristic of the sample

Variables	pathological IT (*n* = 27)	non-pathological IT (*n* = 26)	t/χ^2^; *p*
*Demographics*			
Age, Years	21.37 ± 3.16	20.92 ± 1.57	0.65; *p* =.52
%Women, *n*	66.7, 18	42.3%, 11	3.17; *p* =.07
BMI, Kg/m^2^	20.85 ± 3.63	21.87 ± 2.53	1.70; *p* =.24
Education, Years	13.78 ± 1.34	13.46 ± 1.10	0.93; *p* =.35
Smoking Status, y/n	12 yes, 14 no	12 yes, 15 no	0.16; *p* =.90
Cigarettes per Day, *n*	1.89 ± 2.29	1.92 ± 2.20	0.55; *p* =.95
Alcohol, units/week	3.04 ± 2.00	2.50 ± 1.96	0.09; *p* =.33
Physical Exercise, time/week	0.89 ± 1.01	1.50 ± 1.10	2.10; *p* =.04
*Clinical Assessment*			
PSWQ	61.11 ± 6.92	41.62 ± 5.50	11.61; *p* <.0001
PTQ	36.30 ± 8.48	23.85 ± 7.39	5.68; *p* <.0001
CES-D	25.07 ± 8.23	16.88 ± 8.44	3.54; *p* <.0001
STAI-Y	52.33 ± 10.34	41.92 ± 6.54	4.35; *p* <.0001
IES-R	43.81 ± 16.17	28.15 ± 13.64	3.80; *p* <.0001
OCI-R	20.15 ± 10.79	12.52 ± 7.79	3.08; *p* =.003
RRS	57.41 ± 11.59	45.58 ± 10.92	3.82; *p* <.0001
GAD-7	9.73 ± 4.21	5.33 ± 2.22	4.56; *p* <.0001
Symptom-Related Difficulties^a^	1.35 ± 0.74	0.79 ± 0.41	3.21; *p* =.002
GAD-Q-IV	1.66 ± 0.74	4.30 ± 3.36	3.37; *p* =.001
HR (pre-induction)	101.38 ± 29.82	80.11 ± 22.08	2.82; *p* =.007
HRV (pre-induction)	35.16 ± 11.83	54.59 ± 18.39	4.31; *p* <.0001
*VAS (baseline)*			
Absent mindedness	51.07 ± 24.09	38.57 ± 26.21	1.86; *p* =.07
Repetitiveness	51.79 ± 25.53	33.21 ± 25.83	2.71; *p* =.01
Intrusiveness	55.71 ± 26.45	32.96 ± 23.67	3.36; *p* =.001
Stuckness	41.48 ± 26.56	21.79 ± 21.61	3.02; *p* =.004
MRI-related worries	34.07 ± 21.53	25.93 ± 23.90	1.32; *p* =.19

Mean (SD) are reported for all variables; PSWQ, Penn State Worry Questionnaire, PTQ, Perseverative Thinking Questionnaire; CES-D, Center for Epidemiologic Studies Depression Scale; STAI-Y, State-Trait Anxiety Inventory; IES-R, Impact of Event Scale-Revised; OCI-R, Obsessive Compulsive Inventory-Revised; RRS, Rumination Response Scale; GAD-7, 7-item Generalized Anxiety Disorder Scale; GAD-Q-IV, Generalized Anxiety Disorder Questionnaire-IV; HR, Heart Rate; HRV, Heart Rate Variability; VAS, Visual Analog Scales; MRI, Magnetic Resonance Imaging; Pathological IT, subsample characterized by a dispositional pathological tendency to engage in intrusive thinking; non-pathological IT, subsample characterized by a low dispositional tendency to engage in intrusive thinking

^a^Response to the GAD-7 question: “*How difficult have these problems made it for you to do your work*,* take care of things at home*,* or get along with other people?*

As shown in Table 1, at baseline, the pathological group exhibited (i) higher levels of dispositional intrusive thinking (PTQ, RRS), symptoms of post-traumatic stress, depression, anxiety, and obsessive-compulsive tendencies; (ii) more repetitive, intrusive, and incontrollable thoughts; and (iii) higher HR and lower HRV compared to controls. No between-group differences were observed regarding MRI-related worries.

### Effects of experimental induction on self-reported and autonomic measures

**Fig. 2:**
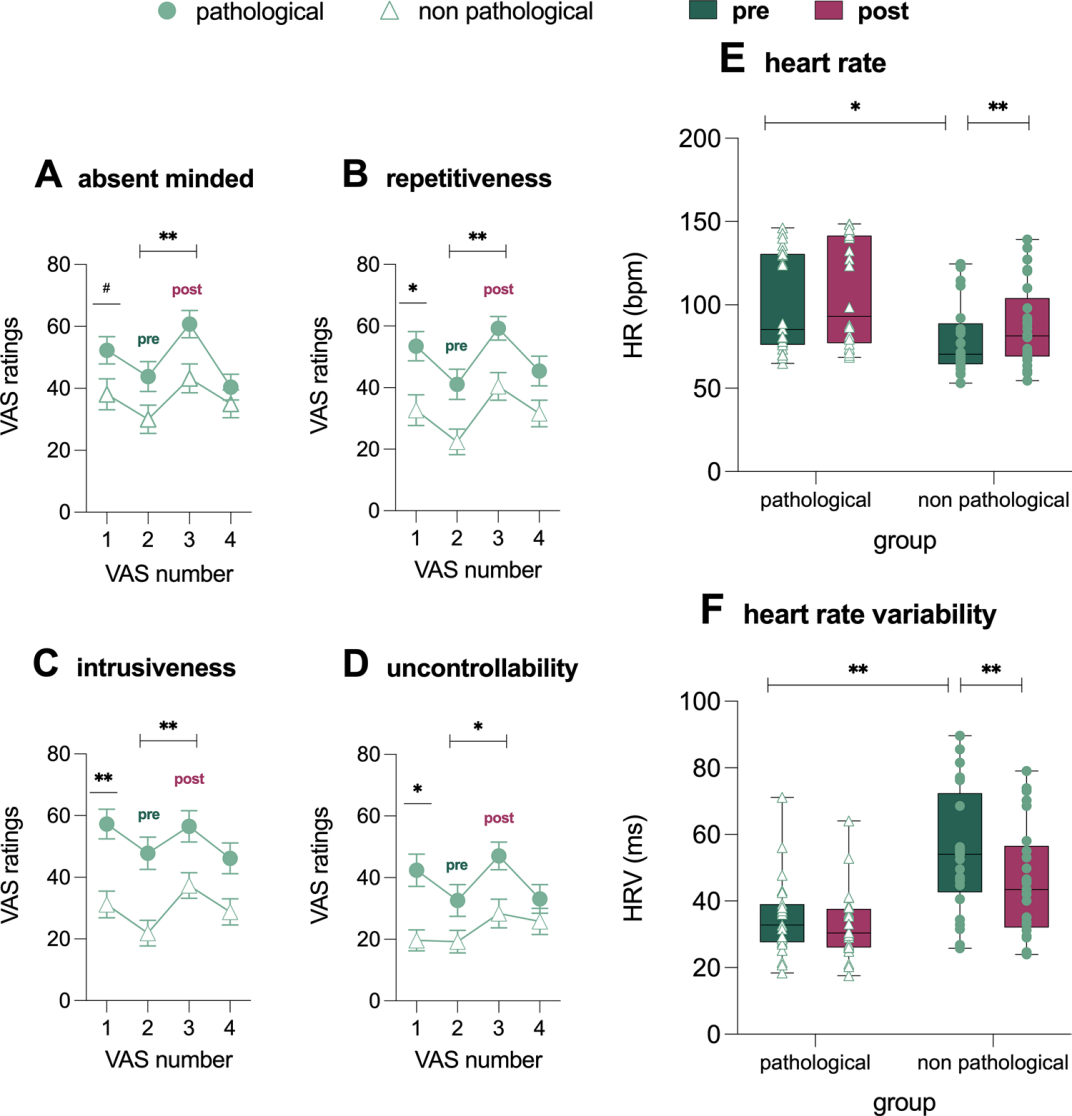
Self-reported levels of absent-mindedness (A), repetitiveness (B), intrusiveness (C), and feeling stuck (D), assessed using Visual Analog Scale (VAS) ratings at four time points: baseline (VAS 1), pre-induction of intrusive thinking (VAS 2), post-induction (VAS 3), and at the end of the paradigm (VAS 4). Dark green and purple box plots represent the mean (thick line) and the 25th and 75th percentiles (thin lines) of pre- and post-induction effects of intrusive thinking on autonomic arousal, as measured by heart rate (HR) (E) and heart rate variability (HRV) (F)

Main effects of Condition and Group were evident for absent mindedness (*F*_1,51_ = 15.80; *p* <.001, *η*_*p*_^*2*^ = 0.24 and *F*_1,51_ = 6.64; *p* =.01, *η*_*p*_^*2*^ = 0.12; Fig. 2A), repetitiveness (*F*_*1,52*_ = 32.08; *p* <.001, *η*_*p*_^*2*^ = 0.38 and *F*_*1,52*_ = 11.66; *p* <.001, *η*_*p*_^*2*^ *=.*18; Fig. 2B), intrusiveness (*F*_*1,52*_ *=* 14.56; *p <*.001, *η*_*p*_^*2*^ = 0.22 and *F*_*1,52*_ = 13.23; *p* <.001, *η*_*p*_^*2*^ *= 0.20*; Fig. 2C), and stuckness (*F*_*1,52*_ *=* 16.65; *p* <.001, *η*_*p*_^*2*^ = 0.24 and *F*_*1,52*_ = 7.78; *p* =.01, *η*_*p*_^*2*^ = 0.13; Fig. 2D), with higher post-induction scores compared to pre-induction and the pathological group reporting higher scores than the non-pathological group (S4 and Table S1). The effects of induction on affective state are reported in the Supplement (S3).

For autonomic measures, significant main effects of *Condition* (*F*_*1,48*_ = 28.00; *p* <.001, *η*_*p*_^*2*^ *=* 0.47) and group (*F*_1,48_ = 5.93; *p* =.014, *η*_*p*_^*2*^ = 0.11) emerged for HR, indicating that the pathological group generally had higher HR than the non-pathological group. A *Condition* by *Group* interaction (*F*_1,48_ = 4.83; *p =*.032, *η*_*p*_^*2*^ = 0.09) was also found. Simple main effect analysis revealed higher HR values during the post-induction phase, primarily driven by the pre-to post-increase in the non-pathological group (Fig. 2E; S4, Table S1).

For HRV, a significant main effect of *Condition* (*F*_*1,48*_ *=* 43.45; *p* <.001, *η*_*p*_^*2*^ *=* 0.48) and *Group* (*F*_*1,48*_ *=* 16.65; *p* <.001, *η*_*p*_^*2*^ *=* 0.26) indicated that HRV was generally lower in the pathological group and during intrusive thinking induction condition. A significant *Condition* by *Group* interaction also emerged (*F*_*1,48*_ = 12.51; *p* <.001, *η*_*p*_^*2*^ *=.*21). HRV values decreased from pre- to post induction, an effect primarily driven by a reduction in the non-pathological group (Fig. 2F; S4, Table S1).

The results of the GLMs for HR and HRV remained unchanged when weekly physical exercise and baseline arousal were included as covariates (S4). Estimated statistical power for the models is reported in S5.

### Effects of experimental induction on GABA and Glx

**Fig. 3:**
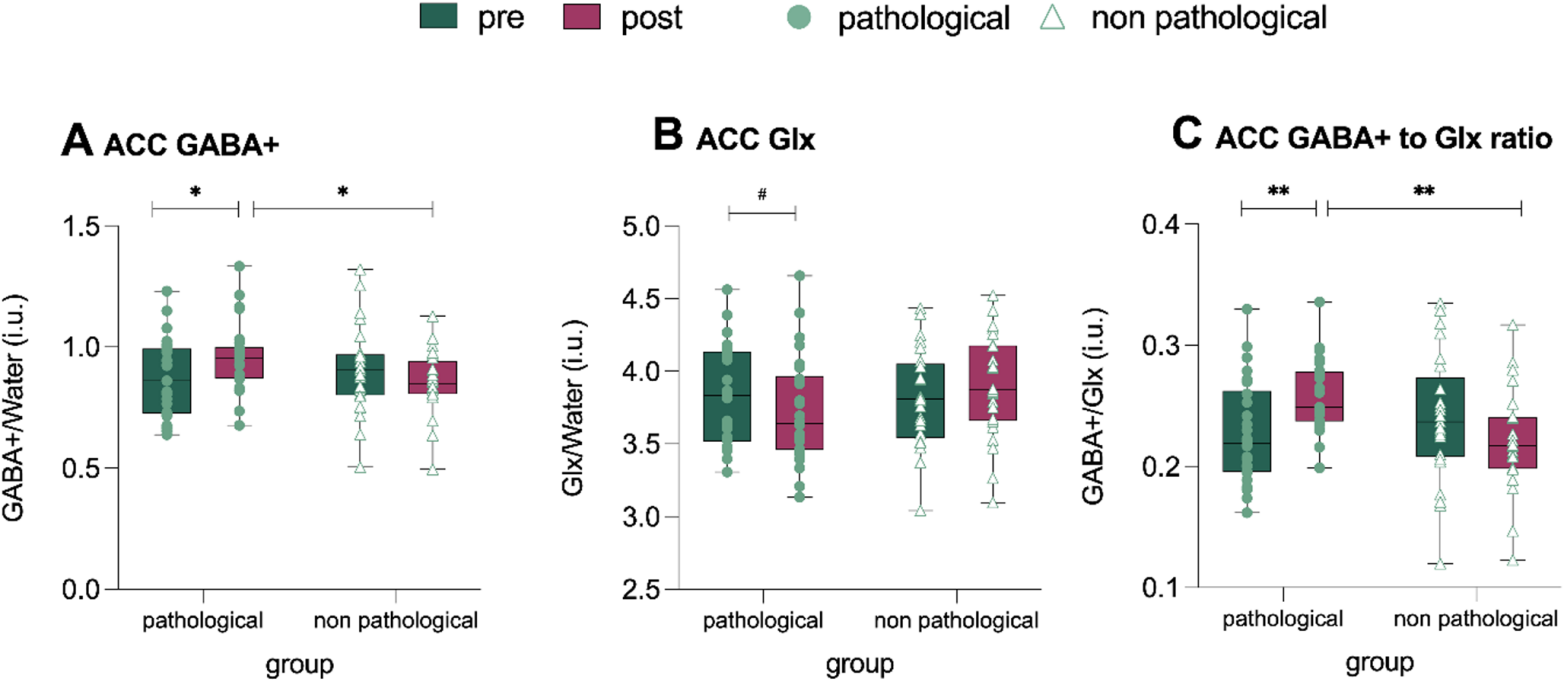
Anterior cingulate cortex (ACC) neurochemical responses during intrusive thinking. Dark green and purple box plots depict mean plots (thick line) and 25th and 75th quartiles (thin lines) of the pre- and- post effects of intrusive thinking induction on GABA+ (A) and Glx (B) neurometabolism, and GABA+/Glx ratio (GABA+/Glx) (C). *Note.* Pathological: subsample characterized by a dispositional pathological tendency to engage in intrusive thinking. Pre: before the induction of intrusive thinking. Post: after the induction of intrusive thinking. Non-pathological: subsample characterized by a low dispositional tendency to engage in intrusive thinking. Error bars indicate mean standard errors. **p <.01; *p <.05; ^#^*p* =.07

In terms of neurochemical effects, a significant *Condition* by *Group* interaction emerged for GABA + levels (*F*_1,47_ = 6.28; *p =*.017, *η*_*p*_^*2*^ = 0.12), with an increase in the pathological group from pre- (0.87 ± 0.16 i.u.) to post-induction (0.96 ± 0.14 i.u.) (*d* = 0.43, *p*_*corrected*_ =.036) and a non-statistically significant decrease in the non-pathological group (pre: 0.90 ± 0.18 i.u., post: 0.86 ± 0.13 i.u., Fig. 3A). A significant difference in ACC GABA + reactivity to the induction was observed between groups (*d* = 0.70, *p* =.022), with the pathological group exhibiting greater ACC GABA + reactivity than the non-pathological group.

The model analyzing Glx revealed a marginally significant *Condition* by *Group* interaction (*F*_1,47_ = 3.19; *p =*.080, *η*_*p*_^*2*^ = 0.06), indicating a trend toward a decrease in the pathological group from pre (3.82 ± 0.34 i.u.) to post-induction (3.73 ± 0.37 i.u.). In contrast, the non-pathological group showed a non-significant increase (pre: 3.81 ± 0.34 i.u., post: 3.90 ± 0.36 i.u., Fig. 3B). ACC Glx reactivity differed marginally between groups (*d* = 0.50, *p* =.082), with the non-pathological group showing a smaller reduction of ACC Glx reactivity compared to pathological group.

For the GABA+/Glx ratio, a significant *Condition* by *Group* interaction emerged (*F*_1,48_ = 12.18; *p =*.001, *η*_*p*_^*2*^ = 0.20) with the pathological group increasing from pre-induction (0.22 ± 0.04 i.u.) to post-induction (0.26 ± 0.03 i.u.) (*d* = 0.66 *p* =.003). No significant changes were observed in the non-pathological groups (pre: 0.24 ± 0.05 i.u. vs. post: 0.23 ± 0.04 i.u.) (Fig. 3C). The ACC GABA+/Glx reactivity to the induction significantly differed between groups (*p* =.001, *d* = 0.98), with the pathological group exhibiting greater ACC GABA+/Glx reactivity than the non-pathological group. Estimated statistical power for the mixed-design GLMs is reported in S5. The results remained unchanged when analyses were conducted controlling for trait anxiety levels (S6).

**Fig. 4:**
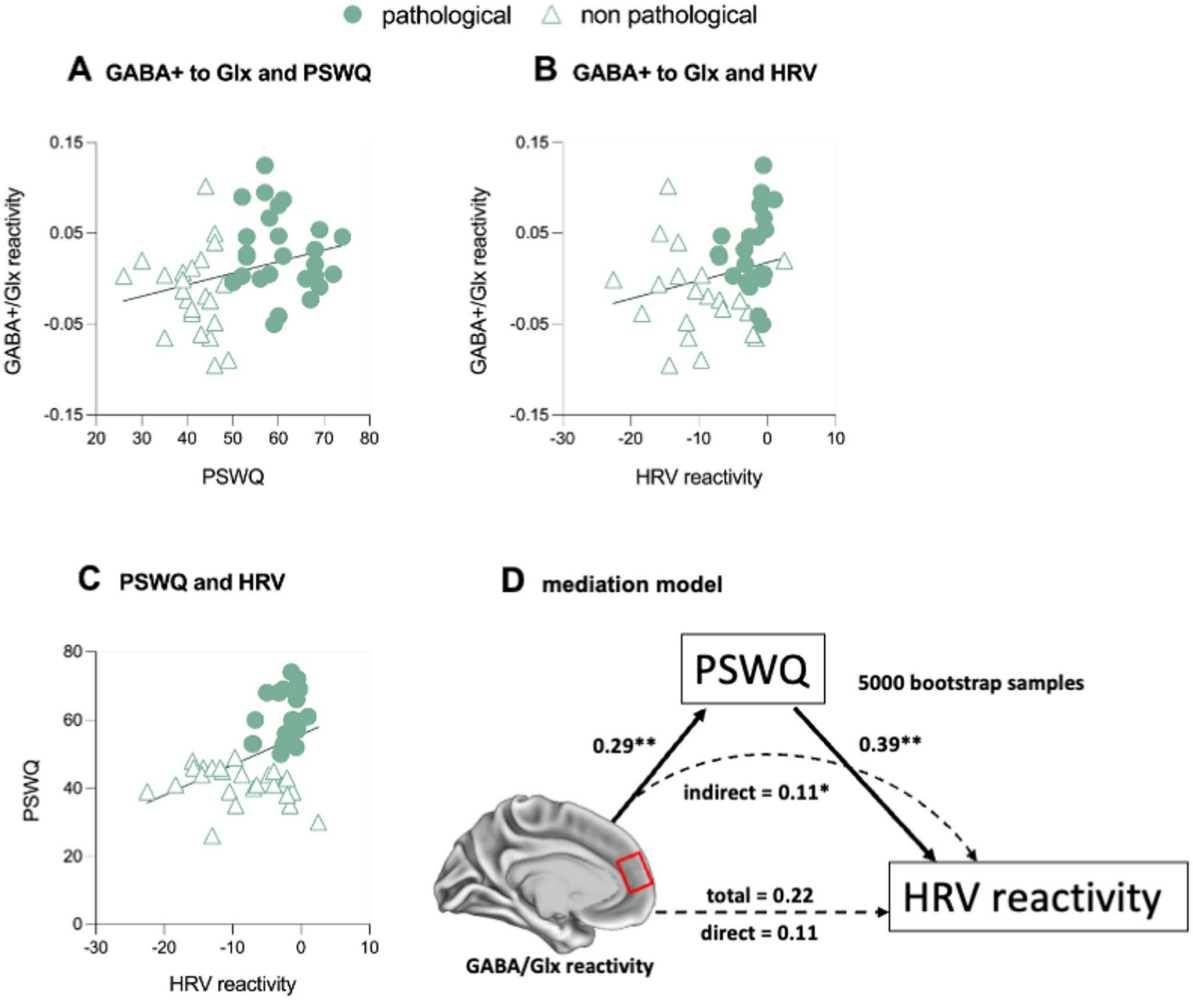
Correlations of GABA+/Glx reactivity to the induction with scores on the Penn State Worry Questionnaire (PSWQ) (A) and heart rate variability (HRV) reactivity to the induction (B). Correlation between scores on the PSWQ and HRV reactivity to the induction (C). Mediation model having GABA+/Glx reactivity within the anterior cingulate cortex (ACC) as the independent variable, HRV reactivity as the dependent variable and PSWQ scores as the mediator (D). Bootstrapping procedure with 5000 bootstrapped samples was used to estimate the 95% confidence interval of the path coefficient effects. Standardized ß-score coefficients are shown (a = 0.29 [0.11, 0.47]; b = 0.39 [0.09, 0.64]; indirect = 0.11 [0.03, 0.23]; direct = 0.11 [-0.18, 0.36]; total = 0.22 [0.11, 0.26]). *Note.* Pathological: subsample characterized by a dispositional pathological tendency to engage in intrusive thinking. Non-pathological: subsample characterized by a low dispositional tendency to engage in intrusive thinking **p =.001, *p =.01

Correlational analyses, corrected for multiple comparisons using the False Discovery Rate (FDR), revealed a positive association between GABA+/Glx ratio reactivity and dispositional intrusive thinking (*r* =.34, *p*_*corrected*_ =.038; Fig. 4A), as well as with HRV reactivity (*r* =.31, *p*_*corrected*_ =.075; Fig. 4B). In contrast, a negative association was observed with HR reactivity (*r* =–.39, *p*_*corrected*_ =.033; Fig. S2). A significant correlation also emerged for dispositional intrusive thinking and HRV reactivity (*r* =.43; *p*_*corrected*_ =.016; Fig. 4C). Based on these correlations, we examined the mediation effects of dispositional intrusive thinking on the relationship between GABA+/Glx reactivity and autonomic arousal (i.e., HRV reactivity) in response to the induction. The 95% confidence intervals for the path coefficients were estimated using a bootstrapping procedure (5000 samples) (Shrout and Bolger 2002). As shown in Fig. 4D, dispositional intrusive thinking mediated the relationship between GABA+/Glx ratio reactivity and HRV reactivity to the induction (indirect effect = 0.11, 95% CI [0.03, 0.23], *p =*.006). The direct effect of dispositional intrusive thinking on HRV reactivity to the induction was not significant after accounting for the mediator (direct effect = 0.11, 95% CI [-0.18, 0.36], *p =*.394, total effect = 0.22, 95% CI [0.11, 0.26], *p =*.233).

### Effects of experimental induction on overall resting state functional connectivity within the central autonomic network

**Fig. 5:**
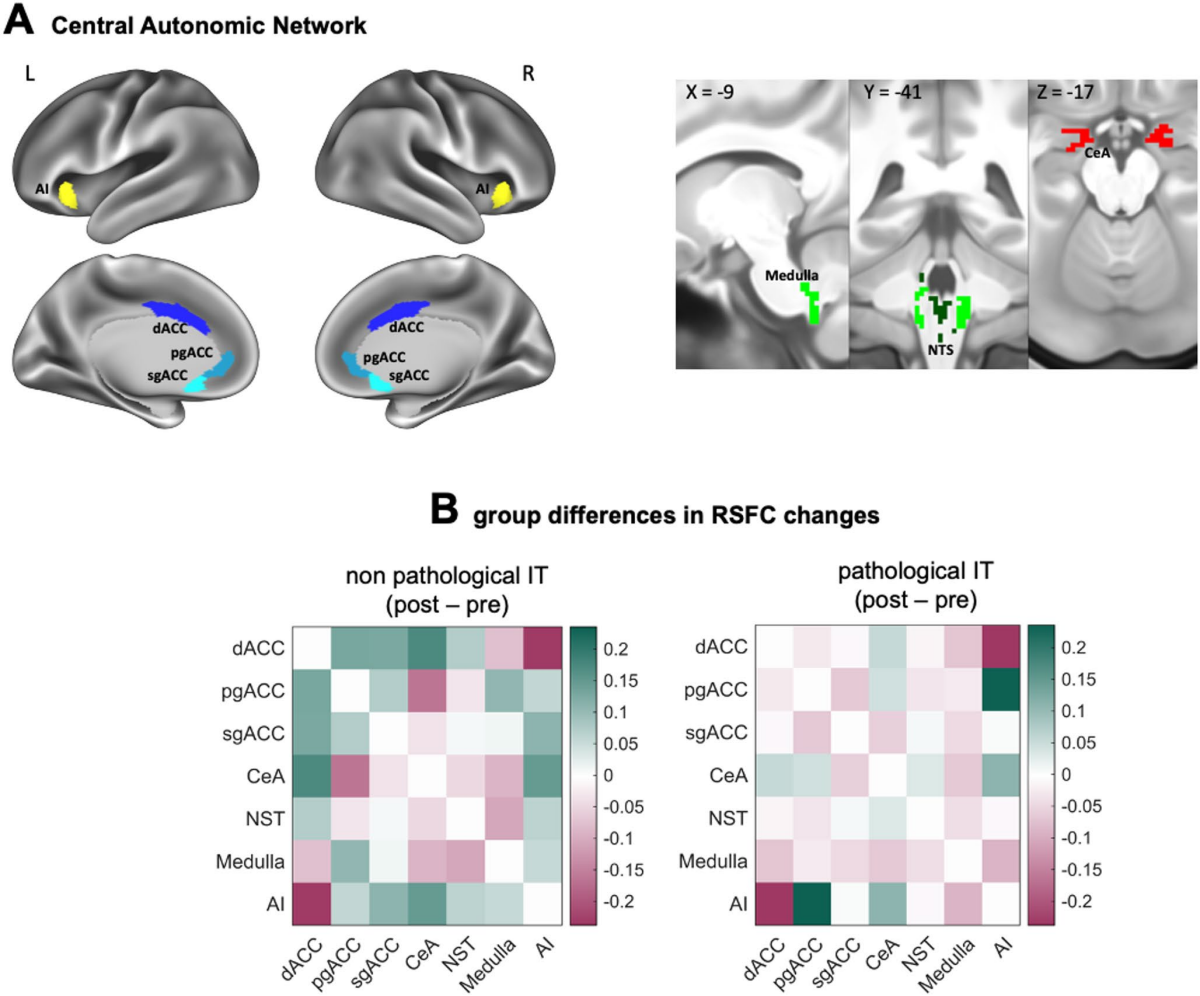
Overall pre-to post-induction changes in resting state functional connectivity (RSFC) within the nodes of Central Autonomic Network (CAN) (A) in individuals with non-pathological and pathological dispositional tendency to engage in intrusive thinking (B). Right-colored bars indicate z-score normalized Pearson correlation values *Note.* Pathological IT: subsample characterized by a dispositional pathological tendency to engage in intrusive thinking. Non-pathological IT: subsample characterized by a low dispositional tendency to engage in intrusive thinking. dACC: dorsal anterior cingulate cortex; pgACC: perigenual anterior cingulate cortex; sgACC: subgenual anterior cingulate cortex; CeA: central nucleus of amygdala; NST: nucleus of solitary tract; AI: anterior insula

A statistically significant adjusted mean difference in overall RSFC within the CAN emerged between the two groups (Wilk’s *λ* = 0.41, *p* =.04; *η*_*p*_^*2*^ *=* 0.59). As shown in Fig. 5, the non-pathological group exhibited increased post-induction RSFC within the CAN compared to the pathological group. Post-hoc MANCOVA did not identify specific pair of nodes contributing to the model, indicating an overall increase in RSFC within the network (Table S2).

## Discussion

Difficulties in interrupting intrusive thoughts and their associated autonomic arousal (e.g., reduced HRV) have been hypothesized to reflect deficits in inhibitory control (e.g., Crespo-García et al. 2022; Ottaviani 2018). The findings of the present study can be summarized as follows: (i) individuals with a higher dispositional tendency toward intrusive thinking exhibited elevated baseline levels of self-reported intrusive thoughts and heightened autonomic arousal; (ii) the experimental induction increased subjective levels of intrusive thinking in both groups; (iii) in the non-pathological group, this increase was accompanied by heightened autonomic arousal and enhanced functional connectivity within CAN regions; (iv) in contrast, the pathological group exhibited reduced functional connectivity and autonomic responses, coupled with a significant pre-to-post induction increase in ACC GABA + concentration.

No significant baseline differences in ACC GABA + or Glx concentrations were observed between the two groups, suggesting that tonic neurometabolic levels may not reliably distinguish pathological from non-pathological intrusive thinking at a trait level. The use of a dimensional rather than diagnosis-based approach may partly account for this finding, which aligns with recent meta-analyses and studies reporting heterogeneous results regarding baseline mPFC GABA + and Glx levels in mood and anxiety disorders (Moriguchi et al. 2019; Simmonite et al. 2023).

Following the experimental induction of intrusive thinking, the non-pathological group showed a trend toward reduced GABA + and increased Glx concentrations, consistent with prior findings on stress-induced changes in mPFC GABA + and Glx levels in healthy humans (Cooper et al. 2021; Hasler et al. 2010; Houtepen et al. 2017; Zwanzger et al. 2013) and rodents (e.g., Bedse et al. 2015; de Groote and Linthorst 2007). This pattern may reflect an adaptive neurochemical response to stress, as suggested by accompanying increases in negative affect, HR and reductions in HRV in this group. These results align with previous findings on PFC Glx responses to an acute stressor only in healthy individuals with low perceived stress levels over the preceding two weeks (Cooper et al. 2021).

In contrast, the pathological group exhibited a significant increase in ACC GABA + levels following the induction, a pattern that has been previously observed in unmedicated individuals with depression (Draganov et al. 2020) and schizophrenia (Reyes-Madrigal et al. 2023). Considering that intrusive thinking can be conceptualized as a form of chronic stress, the current results align with preclinical findings showing that chronic stress enhances inhibition of infralimbic cortex glutamatergic output neurons via increased GABA release, likely due to greater state-dependent GABAergic realese sites on these neurons (McKlveen et al. 2016).

Notably, pre-to post-induction changes in ACC GABA + levels were negatively associated with phasic HR changes and positively associated with HRV changes. This association is consistent with preclinical studies reporting that enhancing GABAergic neurotransmission in the subgenual ACC -using intracerebral Baclofen infusions, a GABA receptor agonist- reduces HR and increases HRV (Wallis et al. 2017), whereas glutamatergic hyperactivation has been associated with the opposite autonomic outcomes (Alexander et al. 2020). These findings support the possible role of GABA ACC in modulating physiological arousal (e.g., Seamans and Floresco 2022).

Additionally, the pathological group showed higher baseline levels of intrusive thinking, HR and lower HRV compared to controls, which is in line with the literature suggesting a predisposition toward hypervigilance and “better safe than sorry” responding in psychopathological profiles (Van den Bergh et al. 2021). One interpretation of the observed increase in ACC GABA + may be that pathological intrusive thinking serves as a maladaptive coping mechanism aimed at regulating autonomic arousal, particularly given the lack of autonomic reactivity observed in this group. This interpretation is compatible with the Contrast Avoidance Model (Newman and Llera 2011), which posits that intrusive thinking persists because it maintains moderate autonomic activation, preventing sudden and distressing surges in arousal, which are highly feared by individuals with anxiety. Although such a strategy may reduce acute distress, chronic reliance on it could contribute to allostatic load and heightened vulnerability to mental and physical health problems (McEwen 2004; McEwen and Akil 2020).

At the large-scale brain network level, if the hypothesis of a GABAergic-mediated mechanism implicated in the avoidance of the negative physiological contrasts underpinning pathological intrusive thinking is plausible, one would expect that the pattern of increased pre- to post-induction GABA + levels seen in the pathological group would be mirrored by reduced pre-to post modulation of the brain network controlling autonomic activity. Supporting this possibility, increased resting state connectivity within CAN regions was observed only in the non-pathological group, namely the group with greater autonomic reactivity. In contrast, the pathological group did not exhibit such modulation. In the work by Makovac and colleagues, the same induction of intrusive thinking led to (i) a decrease in phasic HRV and prefrontal-amygdala RSFC in healthy participants —a pattern similar to that of individuals with GAD at baseline— and, unexpectedly, (ii) a marked increase in prefrontal/cingulum and amygdala RSFC in individuals with GAD (Makovac et al. 2016). Similarly, a previous study reported increased insula-paracingulate cortex RSFC during worry induction (compared to reappraisal) in elderly individuals with GAD (Andreescu et al. 2015). Altogether, these findings suggest that while individuals who habitually engage in intrusive thinking exhibit neural and autonomic patterns indicative of inhibitory control deficits at rest (e.g., Schmitz et al. 2017; Ottaviani et al. 2018), they may employ abnormal levels of inhibitory efforts during bouts of intrusive thinking. While future studies are needed to test this hypothesis and the possible pathophysiological involvement of ACC GABA, an amplified engagement of the prefrontal cortex has also been observed during attempts to control tics in Tourette syndrome (Rae et al. 2020).

In relation to that, interventions such as ketamine, which modulates GABAergic inhibitory inputs on cortical glutamatergic synapses (e.g., Duman et al. 2019; Krystal et al. 2019) or cingulotomy have shown promise for treating internalizing disorders characterized by intrusive thinking, such as treatment-resistant depression and OCD (e.g., Brown et al. 2016; Sanchez et al. 2023; Steele et al. 2008).

The present study has several limitations. A key methodological constraint is the well-known limitation of MRS in quantifying GABA+, namely the potential contamination from co-edited macromolecules. This contamination may artificially inflate or obscure true GABA levels, complicating the interpretation of group differences or condition-related changes. Second, the lack of an active control condition for the intrusive thinking induction (e.g., neutral event recall) limits interpretability. However, our primary aim was to measure neurometabolic changes following intrusive thinking induction relative to participants’ baseline, consistent with prior studies using similar paradigms (e.g., Makovac et al. 2016). Including an active control would have precluded assessment of a true baseline in a within-subject design. Nonetheless, future studies should evaluate whether the observed ACC GABA + reactivity is replicable using designs that include active control conditions. Third, the cross-sectional design restricts our ability to draw causal inferences between intrusive thinking and ACC GABA + levels. Fourth, due to time constraints, we were unable to assess the regional specificity of the neurochemical effects associated with intrusive thinking. Further research is warranted to determine the spatial specificity of the observed state-dependent increase in ACC GABA+.

Keeping these limitations in mind, the present preliminary findings appear to suggest that intrusive thinking may have a state-dependent effects on metabolite concentrations, in particular GABA+, Glx and their ratio, within the ACC, a region implicated in the pathophysiology of several psychiatric conditions (Drevets et al. 1997, 2008; Hamami et al. 2011) for which intrusive thinking and autonomic dysfunctions are central features. Considering that existing evidence in humans typically focuses on resting states or derives from diagnosis-based case-control studies (Sanchez et al. 2023; Weber-Goericke and Muehlhan 2023), this study represents a first step toward a state-dependent investigation of the possible neurochemical pathways implicated in the maintenance of intrusive thinking. Replication in larger, independent cohorts is needed to validate these findings and further clarify the specificity and temporal dynamics of ACC neurometabolic changes associated with intrusive thought processes.

## Electronic Supplementary Material

Below is the link to the electronic supplementary material.

Supplementary Material

## Data Availability

The dataset and scripts generated during the current study are available from the corresponding author upon reasonable request.

## Abbreviations

ACC: Anterior Cingulate Cortex
CAN: Central Autonomic Network
GABA+: Gamma-Aminobutyric Acid macromolecular-contamined
Glx: Glutamate + Glutamine
^1^H-MRS: Proton Magnetic Resonance Spectroscopy
HR: Heart Rate
HRV: Heart Rate Variability
RSFC: Resting state functional connectivity
