# Supplementary material for: State-dependent reactivity of anterior cingulate cortex neurochemistry and downstream autonomic arousal in intrusive thinking
Source: J Neural Transm (Vienna). 2025 Sep 4;132(9):1363–78. doi: 10.1007/s00702-025-02992-2 (PMC12535533; doi:10.1007/s00702-025-02992-2)
Supplement: Supplementary file 1 — Supplementary Material [file 702_2025_2992_MOESM1_ESM.docx]

**State-Dependent Reactivity of Anterior Cingulate Cortex Neurochemistry and Downstream Autonomic Arousal in Intrusive Thinking**

**S1. ^1^H-MRS data images acquisition and preprocessing details**

MEGA-PRESS data were acquired with TE/TR 68/2500 ms; editing pulses were set at 7.5 and 1.9 p.p.m. (Bandwidth 51 Hz). 2048 points were sampled at 1.5 kHz bandwidth. The sequence included VAPOR (VAriable Power Optimized Relaxation delays) water suppression (Bandwidth 75 Hz, Tkác et al., 1999) and Outer Volume Saturation (OVS).

Before MRS scanning, first and second order shim terms were automatically optimized with FASTMAP (Gruetter, 1993), with a target water linewidth below 14 Hz. In case the target was not reached, the voxel was slightly moved away from skull and the procedure repeated. Additionally, MEGA-PRESS transmission power, VAPOR reference flip angle and the last VAPOR delay before the last two OVS blocks (T7, Tkac and Gruetter, 2005) were optimized subject by subject with appropriate scanning cycles.

In each session (pre/post induction), three spectra were acquired (2x64 transients each, plus 4 prescans for magnetization and scanner stabilization). Reference frequency was set at 3 p.p.m. and re-set before the acquisition of each spectrum of each session to compensate for frequency drift. Transients were saved separately for further processing. Unsuppressed water signal was acquired in the same voxel for referencing and eddy currents correction with the same parameters, but TR= 5000 ms.

^1^H-MRS data pre-processing, performed in GANNET, involved: merge of the 3 water suppressed scans acquired on each session, eddy current correction, frame-by-frame frequency alignment by SpecRegHERMES algorithm (Mikkelsen et al., 2018), separation of two sets of subspectra, labelled ON and OFF, obtained respectively with and without the application of an editing pulse at 1.9 ppm, generation of an average difference spectrum (DIFF) data and removal of residual water.

The resulting averages stored in the time domain for each participant both pre- and post-induction were fed to LCModel (Provencher, 2001). After frequency and phase-correction, data were water referenced and quantified in a chemical shift range between 0.2 and 4.0 p.p.m. by linear combination of metabolites from a simulated basis set. The basis set was simulated for 3T with the Python-based VESPA package (https://github.com/vespa-mrs/vespa.io), and included GABA, glutamine (Gln), glutamate (Glu), glutathione (GSH), N-acetylaspartate (NAA), N-acetylaspartylglutamate (NAAG), NAA+NAAG, Glx (Gln+Glu), GSH+Glu+Gln and macromolecules at 0.9 ppm (MM09). Lipid contamination and water suppression were visually checked for each quantified spectrum (Figure S1).

**S2. Resting-state functional MRI data preprocessing**

Preprocessing was performed according to the HCP pipeline as implemented in QuNEX (Ji et al., 2022), with the following steps (Wang et al., 2022): (i) The HCP PreFreeSurfer, FreeSurfer, and PostFreeSurfer pipelines were successively conducted on the T1 MPRAGE and T2 images. This process included correcting the gradient distortion, aligning repeated runs, removing the skull from the image, removing readout distortion, performing bias field correction, registering the image to the standard Montreal Neurological Institute space, and producing tissue maps and surface files for pial and white matter for each participant, followed by down-sampling and registering surface files; (ii) after the structural preprocessing was complete, the HCP fMRIVolume and fMRISurface pipelines were used on the functional and resting images. This process included removing spatial distortions, realigning the volumes to compensate for participant motion, registering the fMRI data to the structural information, reducing the bias field, normalizing the 4D image to a global mean, masking the data, and transferring the time series from the volume into the CIFTI standard space (Glasser et al., 2013).

Functional data were then additionally preprocessed through QuNex's internal functions (i.e., *extract_nuisance*, *preprocess_bold*) to remove the artifactual noise (motion, ventricular, white matter, respiratory, and pulse signals) and their first derivatives by regressing them out of the BOLD signal. A bandpass filter of 0.01–0.1 Hz was applied to mitigate the contribution of the nuisance signal in the BOLD timeseries. fMRI data were downsampled to the whole-brain cortical and subcortical atlas Cole-Anticevic Brain-wide network partition (Ji et al., 2019), yielding 718 fMRI time series for each participant’s pre- and post-induction conditions.

**S3. Effects of the experimental induction of intrusive thinking on visual analogue mood scale ratings**

The mixed design having the visual analog scales (VASs) assessing pre- to post-induction changes in affective state yielded main effects of condition for levels of i) happiness (*F_1,45_ = 37.07; p < .001, η_p_^2^ = .45;* pre: 53.19 ± 16.62 vs post: 38.51 ± 16.68) and calmness (*F_1,44_ = 23.07; p < .001, η_p_^2^ = .34;* pre: 63.26 ± 21.61 vs post: 47.83 ± 19.99), with a decrease in both groups; ii) anger (*F_1,51_ = 29.69; p < .001, η_p_^2^ = .37* pre: 13.96 ± 20.41 vs post: 27.55 ± 26.88) and anxiety (*F_1,51_ = 17.02; p < .001, η_p_^2^ = .25;* pre: 27.88 ± 25.23 vs post: 38.27 ± 26.69), with an increase in both groups; and iii) no significant changes in levels of tiredness (pre: 48.65 ± 21.42 vs post: 48.46 ± 21.91) or MRI-related worries (pre: 23.77 ± 21.32 vs post: 23.40 ± 21.66). Overall, the induction of intrusive thinking worsened the affective state of participants, irrespective of their habitual tendency to engage in this dysfunctional coping strategy (Table S2).

**S4. Additional statistical details on intrusive thinking induction effects on VAS ratings and autonomic indices**

For absent mindedness, post-induction scores were significantly higher comparted to pre-induction (*d* = 0.58, *p_corrected_* < .001). Overall, the pathological group reported higher levels than the non-pathological group (*d* = 0.74, *p_corrected_* = .023). For repetitiveness, post-induction scores were significantly higher than pre-induction (*d* = 0.71, *p_corrected_* < .001), with the pathological group showing higher scores than the non-pathological group (*d* = 0.85, *p_corrected_* < .001). For intrusiveness, scores significantly increased from pre- to post-induction (*d* = 0.44, *p_corrected_* = .001). The pathological group reported higher levels than the non-pathological group overall (*d* = 0.79, *p_corrected_* = .001). For uncontrollability, post-induction scores were significantly higher compared to pre-induction (*d* = 0.49, *p_corrected_* < .001), with higher overall scores observed in the pathological group (*d* = 0.78, *p_corrected_* = .015).

Regarding autonomic indices, post-induction HR was significantly higher than pre-induction levels (*d* = 0.19, *p_corrected_* < .001). The pathological group showed generally higher HR compared to the non-pathological group (*d* = 0.67, *p_corrected_* = .029). However, simple main effects analyses indicated that the post-induction HR increase was primarily driven by the non-pathological group.

For HRV, post-induction levels were significantly lower than pre-induction (*d* = 0.33, *p_corrected_* < .001). The pathological group displayed overall lower HRV compared to the non-pathological group (*d* = 0.90, *p_corrected_* < .001). Simple main effects analyses revealed that the group difference was largely driven by a significant pre-to-post HRV decrease in the pathological group (*d* = 0.48, *p_corrected_* < .001).

To account for individual differences in baseline arousal, we computed reactivity scores for HR and HRV using the formula (post – pre)/pre, consistent with the approach used for neurochemical indices (GABA+, Glx, and GABA+/Glx ratio). Group differences were then assessed using independent-samples *t*-tests. Results indicated significantly reduced HRV reactivity in the pathological group (*t* = –4.89, *p_corrected_* < .001, 95% *CI* [–9.42, –3.89]), but no significant group difference in HR reactivity (*t* = 1.61, *p* = .113, 95% *CI* [–0.70, 6.37]). These findings suggest that group differences in HRV responses to the induction persist even after accounting for baseline arousal levels.

The results for HR and HRV were reanalyzed controlling for weekly physical exercise. For HR, a significant main effect of *Condition* (*F*₁,₄₈ = 16.67; *p* < .001; *ηp²* = .27) and *Group* (*F*₁,₄₈ = 6.57; *p* = .013; *ηp²* = .12) emerged, while the *Condition × Group* interaction was not significant (*F*₁,₄₈ = 2.11; *p* = .156; *ηp²* = .04). No significant main effect or interaction involving *Physical Exercise* was observed (*F*₁,₄₈ = 0.37; *p* = .547; *ηp²* = .01; *F*₁,₄₈ = 0.01; *p* = .914; *ηp²* = .00).

For HRV, significant main effects of *Condition* (*F*₁,₄₈ = 15.10; *p* < .001; *ηp²* = .25) and *Group* (*F*₁,₄₈ = 16.20; *p* < .001; *ηp²* = .26), as well as a significant *Condition × Group* interaction (*F*₁,₄₈ = 15.48; *p* < .001; *ηp²* = .25), were found. While there was no significant main effect of *Physical Exercise* (*F*₁,₄₈ = 1.66; *p* = .203; *ηp²* = .03), a significant *Condition × Physical Exercise* interaction emerged (*F*₁,₄₈ = 4.68; *p* = .035; *ηp²* = .09).

**S5. Estimated statistical power for the mixed-design ANOVA models**

The average statistical power across all significant effects was 0.90 [95% CI: 0.68–1.00], indicating adequate power to detect effects of the observed magnitude.

Absent minded. 1- *β* (Group effect): 0.75; 1- *β* (Condition effect): 0.99

Repetitiveness. 1- *β* (Group effect): 0.92; 1- *β* (Condition effect): 0.99

Intrusiveness. 1- *β* (Group effect): 0.95; 1- *β* (Condition effect): 0.99

Uncontrollability. 1- *β* (Group effect): 0.95; 1- *β* (Condition effect): 0.97

HR. 1- *β* (Group effect): 0.75; 1- *β* (Condition effect): 1.00; 1- *β* (Condition x Group): 0.68

HRV. 1- *β* (Group effect): 0.95; 1- *β* (Condition effect): 1.00; 1- *β* (Condition x Group): 0.95

GABA+. 1- *β* (Condition x Group): 0.72

GABA+/Glx ratio. 1- *β* (Condition x Group): 0.94

**S6. Effects of experimental induction on GABA and Glx controlling for trait levels of anxiety**

To account for individual differences in trait anxiety, *STAI-Y* scores were included as a covariate in mixed-design ANOVAs examining *GABA+* and the *GABA+/Glx* ratio as outcome variables.

For *GABA+*, the analysis revealed a significant main effect of Condition (*F*_1,47_ = 8.12, *p* = .006, *ηp*² = .29, 1–*β* = 0.96) and a significant Condition × Group interaction (*F*_1,47_ = 13.43, *p* < .001, *ηp*² = .13, 1–*β* = 0.96), but no significant main effect involving trait anxiety (*F*_1,47_ = 1.85, *p* = .180, *ηp*² = .03). Simple effects analyses indicated patterns consistent with those observed in the analyses without the covariate: the pathological group showed a significant increase from pre- (*M* = 0.86 ± 0.16 i.u.) to post-induction (*M* = 0.95 ± 0.14 i.u.; *p*_corrected_ = .002), while the non-pathological group exhibited a significant decrease (pre: 0.90 ± 0.13 i.u.; post: 0.85 ± 0.18 i.u.; *p*_corrected_ = .018).

Similarly, for the *GABA+/Glx* ratio, there was a significant main effect of Condition (*F*_1,47_ = 6.73, *p* = .012, *ηp*² = .12, 1–*β* = 0.76) and a significant Condition × Group interaction (*F*_1,47_  = 19.23, *p* < .001, *ηp*² = .14, 1–*β* = 0.81), with no significant main effect of trait anxiety (*F*_1,47_  = 0.17, *p* = .677, *ηp*² = .00, 1–*β* = 0.07). Again, the pattern of results was consistent with previous analyses: the pathological group showed a significant increase from pre- (*M* = 0.22 ± 0.04 i.u.) to post-induction (*M* = 0.25 ± 0.02 i.u.; *p*_corrected_ < .001), while the non-pathological group showed a significant decrease (pre: 0.23 ± 0.05 i.u.; post: 0.22 ± 0.04 i.u.; *p*_corrected_ = .026).

**Table S1.** Mean and standard deviation (±) for the main effects of the experimental induction of intrusive thinking on self-reported and autonomic measures.

|  | **Pre-induction** | | |  | | **Post-induction** | |
| --- | --- | --- | --- | --- | --- | --- | --- |
| **Variable** | | **pathological** | **non-pathological** |  | **pathological** | | **non-pathological** |
| Absent minded | | 43.85 ± 24.67 | 30.00 ± 23.66 |  | 60.74 ± 23.03 | | 43.24 ± 23.4 |
| Repetitiveness | | 41.11 ± 25.47 | 22.40 ± 20.76 |  | 59.26 ± 19.99 | | 40.38 ± 23.24 |
| Intrusiveness | | 47.78 ± 27.36 | 21.92 ± 21.36 |  | 56.54 ± 25.91 | | 37.31 ± 21.46 |
| Stuck | | 32.59 ± 26.83 | 19.23 ± 18.75 |  | 47.04 ± 25.50 | | 28.40 ± 23.39 |
| MRI worries | | 26.67 ± 21.48 | 20.77 ± 21.15 |  | 26.30 ± 20.97 | | 20.38 ± 22.36 |
| HR (bpm) | | 101.39 ± 29.83 | 80.11 ± 22.08 |  | 106.71 ± 31.86 | | 87.16 ± 24.79 |
| HRV (ms) | | 35.16 ± 11.84 | 54.59 ± 18.39 |  | 32.78 ± 10.32 | | 24.79 ± 16.83 |

*Note.* HR, Heart Rate; HRV = Heart Rate Variability; MRI = Magnetic Resonance Imaging; Pathological, sample characterized by a dispositional pathological tendency to engage in intrusive thinking; non-pathological, sample characterized by a low dispositional tendency to engage in intrusive thinking. See Figure 2 for a graphical representation.

**Table S2.** Mean and standard error (±) for the main effects of the experimental induction of intrusive thinking on pre-post resting-state functional connectivity changes between the nodes of the central autonomic network (CAN), controlling for biological sex.

| **CAN nodes** | | **pathological** | **non-pathological** | |
| --- | --- | --- | --- | --- |
| dACC and pgACC | -0.46 ± 1.47 | | | -3.48 ± 1.82 |
| dACC and sgACC | 0.47 ± 0.94 | | | -0.36 ± 0.29 |
| dACC and sgACC | 0.89 ± 1.12 | | | -0.95 ± 0.78 |
| dACC and Medulla | 4.37 ± 7.48 | | | -1.85 ± 0.56 |
| dACC and NTS | -0.46 ± 0.53 | | | -2.74 ± 1.32 |
| dACC and AI | -1.2 ± 0.53 | | | -7.66 ± 7.7 |
| pgACC and sgACC | 0.38 ± 0.18 | | | 0.74 ± 0.33 |
| pgACC and CeA | -1.1 ± 0.6 | | | -2.75 ± 1.77 |
| pgACC and Medulla | -2 ± 1.15 | | | -3.44 ± 2.47 |
| pgACC and NTS | -4.76 ± 5.35 | | | -0.56 ± 0.78 |
| pgACC and AI | 0.08 ± 0.07 | | | -0.03 ± 0.07 |
| sgACC and CeA | 0.03 ± 1.14 | | | -0.43 ± 0.43 |
| sgACC and Medulla | -303.6 ± 302.1 | | | 16.56 ± 17.7 |
| sgACC and NTS | -0.48 ± 0.5 | | | -0.68 ± 0.44 |
| sgACC and AI | 0.4 ± 0.2 | | | 7.93 ± 7.84 |
| CeA and Medulla | -0.1 ± 0.63 | | | 7.39 ± 6.89 |
| CeA and NTS | 4.61 ± 5.37 | | | 1.69 ± 2.4 |
| CeA and AI | -0.7 ± 0.39 | | | 27.67 ± 27.18 |
| Medulla and NTS | 0.46 ± 0.44 | | | 0.31 ± 0.29 |
| Medulla and AI | -6.96 ± 4.56 | | | -2.27 ± 1.26 |
| NTS and AI | 1.05 ± 0.97 | | | 6.39 ± 5.56 |

*Note.* dACC, dorsal anterior cingulate cortex; pgACC, perigenual anterior cingulate cortex; sgACC, subgenual anterior cingulate cortex; CeA, central nucleus of amygdala; NST, nucleus of solitary tract; AI, anterior insula; pathological, sample characterized by a dispositional pathological tendency to engage in intrusive thinking; non-pathological, sample characterized by a low dispositional tendency to engage in intrusive thinking. See Figure 5 for a graphical representation.

**Figure S1.** Each quantified spectrum in the current sample for the pre- (PRE) and post-induction (POST) conditions of intrusive thinking and groups, separately. Stacked plot spectra and stacked plot GABA+ fit are depicted.

*
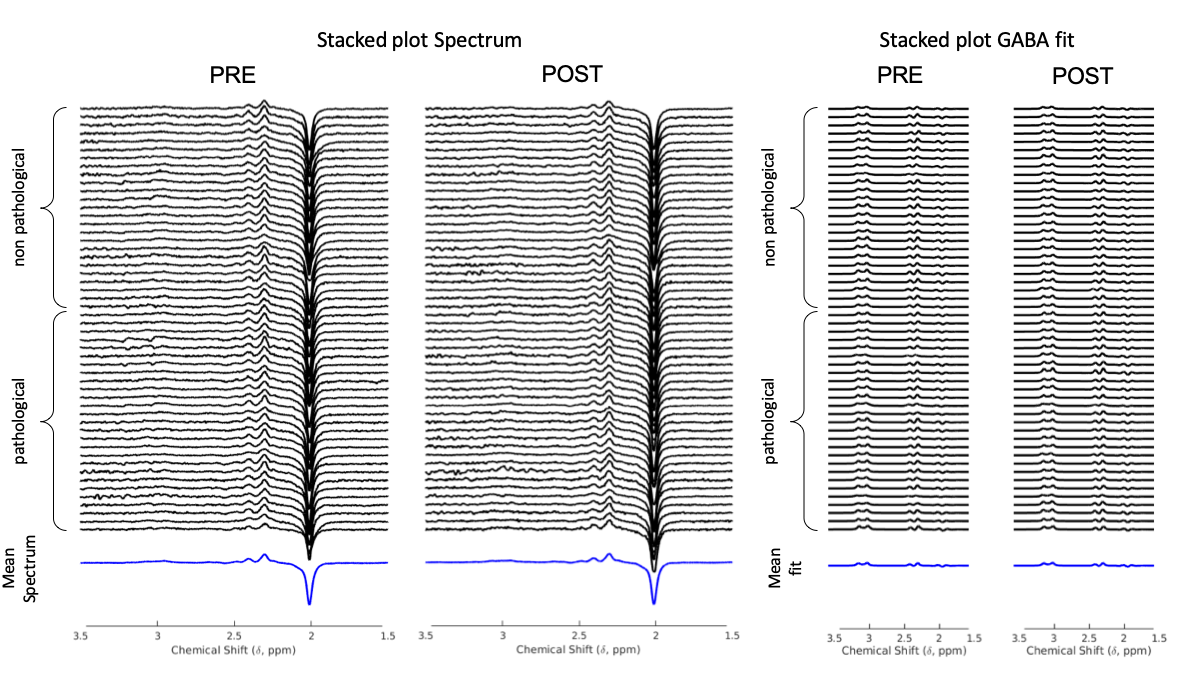
*

*Note.* Pathological, sample characterized by a dispositional pathological tendency to engage intrusive thinking; non-pathological, sample characterized by a low dispositional tendency to engage in intrusive thinking.

**Figure S2.** Correlations between GABA+/Glx reactivity and heart rate reactivity to the experimental induction of intrusive thinking.

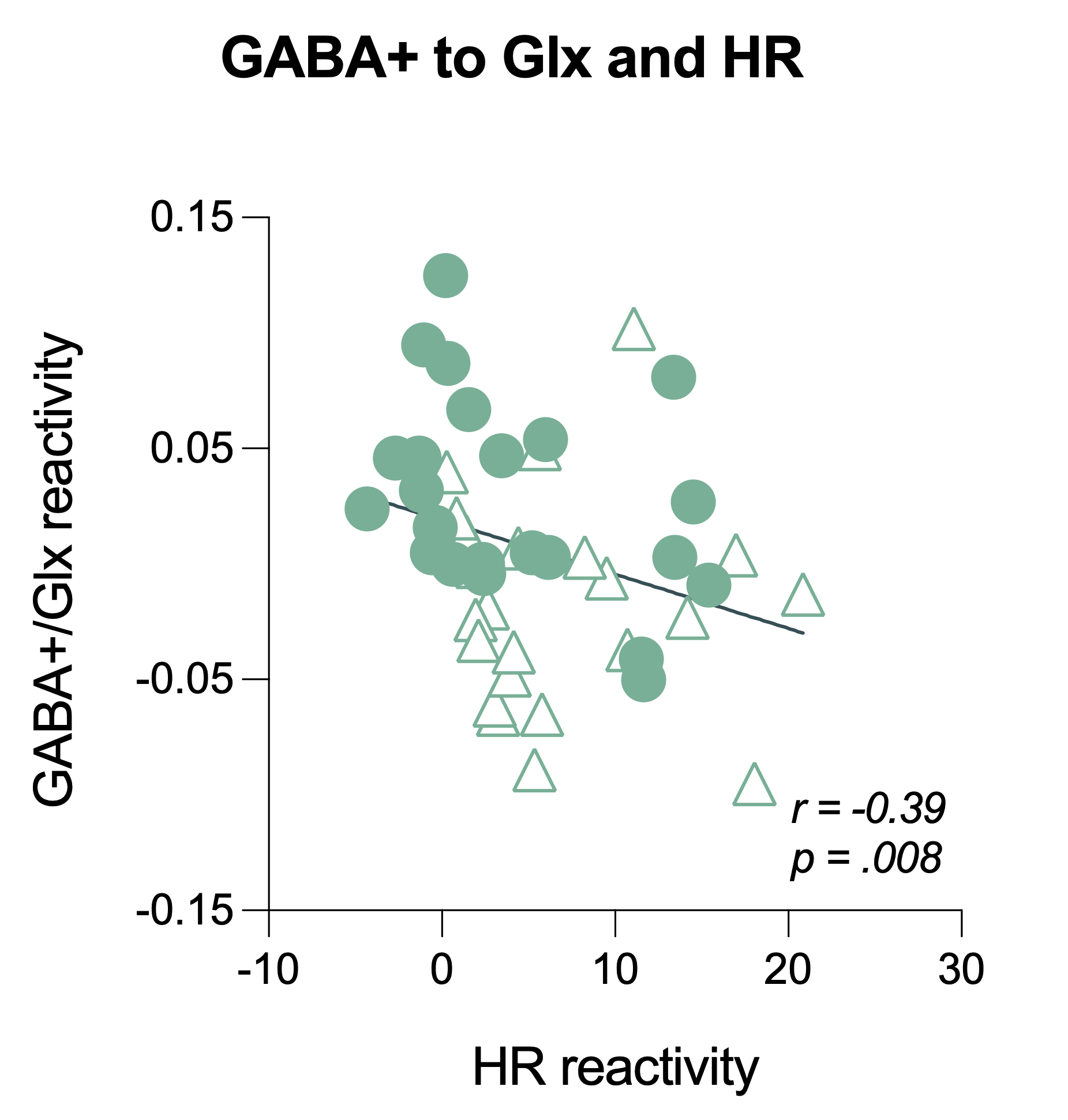


*Note.* Pathological, sample characterized by a dispositional pathological tendency to engage in intrusive thinking; non-pathological, sample characterized by a low dispositional tendency to engage in intrusive thinking.

**References**

Glasser, M. F., Sotiropoulos, S. N., Wilson, J. A., Coalson, T. S., Fischl, B., Andersson, J. L., Xu, J., Jbabdi, S., Webster, M., Polimeni, J. R., Van Essen, D. C., & Jenkinson, M. (2013). The minimal preprocessing pipelines for the Human Connectome Project. *NeuroImage*, *80*, 105–124. <https://doi.org/10.1016/j.neuroimage.2013.04.127>

Ji, J. L., Demšar, J., Fonteneau, C., Tamayo, Z., Pan, L., Kraljič, A., Matkovič, A., Purg, N., Helmer, M., Warrington, S., Winkler, A., Zerbi, V., Coalson, T. S., Glasser, M. F., Harms, M. P., Sotiropoulos, S. N., Murray, J. D., Anticevic, A., & Repovš, G. (2022). *QuNex – An Integrative Platform for Reproducible Neuroimaging Analytics* [Preprint]. Neuroscience. <https://doi.org/10.1101/2022.06.03.494750>

Ji, J. L., Spronk, M., Kulkarni, K., Repovš, G., Anticevic, A., & Cole, M. W. (2019). Mapping the human brain’s cortical-subcortical functional network organization. *NeuroImage*, *185*, 35–57. <https://doi.org/10.1016/j.neuroimage.2018.10.006>

Mikkelsen, M., Saleh, M. G., Near, J., Chan, K. L., Gong, T., Harris, A. D., Oeltzschner, G., Puts, N. A. J., Cecil, K. M., Wilkinson, I. D., & Edden, R. A. E. (2018). Frequency and phase correction for multiplexed edited MRS of GABA and glutathione. *Magnetic Resonance in Medicine*, *80*(1), 21–28. <https://doi.org/10.1002/mrm.27027>

Provencher, S. W. (2001). Automatic quantitation of localized in vivo1H spectra with LCModel. *NMR in Biomedicine*, *14*(4), 260–264. <https://doi.org/10.1002/nbm.698>

Tkáć, I., & Gruetter, R. (2005). Methodology of1H NMR spectroscopy of the human brain at very high magnetic fields. *Applied Magnetic Resonance*, *29*(1), 139–157. <https://doi.org/10.1007/BF03166960>

Tkác, I., Starcuk, Z., Choi, I.Y., Gruetter, R. (1999). In vivo1H NMR spectroscopy of rat brain at 1 ms echo time. *Magnetic Resonance in Medicine*, *41*(4), 649–656. [https://doi.org/10.1002/(SICI)1522-2594(199904)41:4<649::AID-MRM2>3.0.CO;2-G](https://doi.org/10.1002/(SICI)1522-2594(199904)41:4%3c649::AID-MRM2%3e3.0.CO;2-G)

Wang, S., Zhang, Y., Zhang, X., Sun, J., Lin, N., Zhang, J., Zong, C., 2022. An fMRI Dataset for Concept Representation with Semantic Feature Annotations. *Scientific Data*, *9*, 721. https://doi.org/10.1038/s41597-022-01840-2
